# Supplementary material for: The causal role of circulating inflammatory markers in osteoporosis: a bidirectional Mendelian randomized study
Source: Front Immunol. 2024 Jul 18;15:1412298. doi: 10.3389/fimmu.2024.1412298 (PMC11291241; doi:10.3389/fimmu.2024.1412298)
Supplement: Supplementary file 1 [file DataSheet_1.zip › supplementary material/CIM-OSTEOPOROSIS /GCST90274846.csv_forest.pdf]

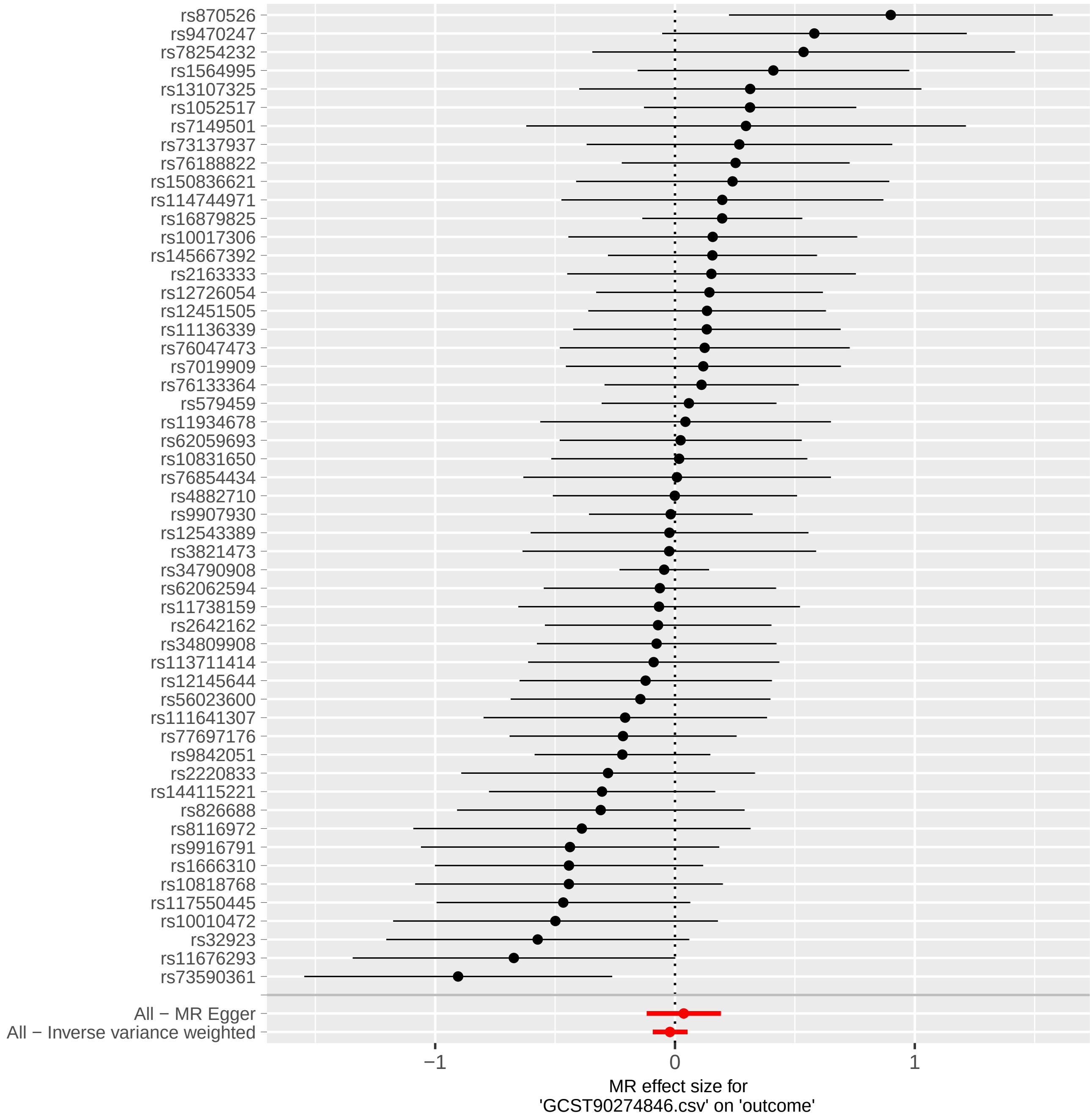
rs870526
rs9470247
rs78254232
rs1564995
rs13107325
rs1052517
rs7149501
rs73137937
rs76188822
rs150836621
rs114744971
rs16879825
rs10017306
rs145667392
rs2163333
rs12726054
rs12451505
rs11136339
rs76047473
rs7019909
rs76133364
rs579459
rs11934678
rs62059693
rs10831650
rs76854434
rs4882710
rs9907930
rs12543389
rs3821473
rs34790908
rs62062594
rs11738159
rs2642162
rs34809908
rs113711414
rs12145644
rs56023600
rs111641307
rs77697176
rs9842051
rs2220833
rs144115221
rs826688
rs8116972
rs9916791
rs1666310
rs10818768
rs117550445
rs10010472
rs32923
rs11676293
rs73590361
All − MR Egger
All − Inverse variance weighted
−1
0
1
MR effect size for
'GCST90274846.csv' on 'outcome'
